# Endothelium-derived fibronectin regulates neonatal vascular morphogenesis in an autocrine fashion

**DOI:** 10.1007/s10456-017-9563-8

**Published:** 2017-06-30

**Authors:** Christopher J. Turner, Kwabena Badu-Nkansah, Richard O. Hynes

**Affiliations:** 1 Howard Hughes Medical Institute, Koch Institute for Integrative Cancer Research, Massachusetts Institute of Technology, 77 Massachusetts Ave, 76-361, Cambridge, MA 02139 USA; 2 Present Address: University of Suffolk, James Hehir Building, University Avenue, Ipswich, Suffolk IP3 0FS UK; 3 Present Address: Duke University Medical Center, 307 Research Drive, Durham, NC 27710 USA

**Keywords:** Fibronectin, Angiogenesis, Integrins, EIIIA, EIIIB, Autocrine

## Abstract

Fibronectin containing alternatively spliced EIIIA and EIIIB domains is largely absent from mature quiescent vessels in adults, but is highly expressed around blood vessels during developmental and pathological angiogenesis. The precise functions of fibronectin and its splice variants during developmental angiogenesis however remain unclear due to the presence of cardiac, somitic, mesodermal and neural defects in existing global fibronectin KO mouse models. Using a rare family of surviving EIIIA EIIIB double KO mice, as well as inducible endothelial-specific fibronectin-deficient mutant mice, we show that vascular development in the neonatal retina is regulated in an autocrine manner by endothelium-derived fibronectin, and requires both EIIIA and EIIIB domains and the RGD-binding α5 and αv integrins for its function. Exogenous sources of fibronectin do not fully substitute for the autocrine function of endothelial fibronectin, demonstrating that fibronectins from different sources contribute differentially to specific aspects of angiogenesis.

**Electronic supplementary material:**

The online version of this article (doi:10.1007/s10456-017-9563-8) contains supplementary material, which is available to authorized users.

## Introduction

In addition to providing structural strength and elasticity to blood vessels, the extracellular matrix (ECM) provides instructional signals that control the development, patterning, and stability of the vasculature [1]. The ECM achieves this, in part, by binding and regulating the distribution and activity of growth factors such as the vascular endothelial growth factors (VEGFs), platelet-derived growth factor (PDGFs), fibroblast growth factor (FGFs) and transforming growth factor-β (TGF-β) [2]. The ECM also regulates vascular development by directly binding and conveying both biochemical and biomechanical signals through integrin receptors [3]. Integrins comprise a family of heterodimeric adhesion receptors that contains 16α and 8β subunits that associate to form 24 different receptors that bind to the ECM with distinct yet often overlapping specificities. Previous studies have shown that the interaction of integrins with the ECM is essential for endothelial cell (EC) adhesion and, as a consequence, regulates EC proliferation, migration, and the sprouting of new vessels [4]. Indeed, concentration gradients of immobilised ECM proteins have been shown to control both the direction and speed of EC migration in the absence of chemokines in vitro [5, 6]. Furthermore, ECM-integrin interactions have been shown to regulate EC junction formation, vessel stability and integrity [7], cell polarity and vessel lumen formation [8].

One of the most extensively studied ECM proteins involved in vascular development is the glycoprotein fibronectin (Fn) [9]. Fibronectin is a modular protein consisting of type I, II, and III repeating units and is alternatively spliced to exclude extra EIIIA and EIIIB domains, and portions of the variable (V or IIICS) domain. Fibronectin is found only in vertebrates with an endothelium-lined vasculature [10] and is expressed around early embryonic vessels before the presence of other basement membrane or structural ECM proteins [11, 12]. Interestingly, Fn containing EIIIA and EIIIB domains is highly expressed around angiogenic vessels [13, 14], but is largely absent from mature quiescent vessels in adults until subjected to injury or low or disturbed flow [15, 16], suggesting that both domains are required for the development and remodelling of the vasculature.

The functional importance of fibronectin can be seen from several genetic studies [9]. Global deletion of *Fn* leads to early embryonic lethality due to severe neural, mesodermal, cardiac and vascular defects in mice [12, 17, 18]. Individually EIIIA-null and EIIIB-null mice are viable, fertile, and lack reported defects in either developmental or tumour angiogenesis [19, 20], however mice lacking both EIIIA and EIIIB domains die around E10.5 with multiple developmental and cardiovascular defects [21]. Interestingly, just as observed in *Fn*-null mice and zebrafish (*natter1* mutants), the severity of the defects in EIIIA EIIIB double KO mice varies with genetic background, suggesting the presence of a genetic modifier(s) [21–23]. The RGD motif within Fn, recognised by α5β1 and αv integrins (i.e. αvβ1, αvβ3, αvβ5, αvβ6 and αvβ8), is also essential for embryonic development. Replacement of the RGD by an inactive RGE motif leads to neural, somitic and cardiovascular defects [17, 24], while mice lacking endothelial expression of α5 and αv integrins, the major RGD-binding receptors expressed on ECs, die around E14.5 with defects in the development of the heart, great vessels and lymphatic vasculature [25, 26].

More recently, Fn secreted and assembled ahead of the developing vasculature by astrocytes has been shown to guide EC migration by binding and presenting VEGF to EC tip cells [27], while Fn, including contributions from plasma, has been shown to promote tumour angiogenesis through increased retention of VEGF in the tumour environment [28]. The precise role of endothelial-derived Fn in developmental angiogenesis however remains unclear. Fn-depleted ECs display defects in angiogenic assays in vitro [29], however postnatal deletion of endothelial *Fn* fails to inhibit tumour angiogenesis in mice [30].

To investigate further the role of fibronectin and its alternatively spliced EIIIA and EIIIB domains in developmental angiogenesis in the absence of heart, great vessel, somite, or neural crest defects, we have analysed in detail the postnatal growth and patterning of blood vessels within the retinas of inducible global *Fn* KO mice, EIIIA and EIIIB double KO mice, and inducible endothelial-specific *Fn* mutants. Our results show that vessel outgrowth, branching, sprouting and stability are regulated to a significant degree by EC-derived EIIIA^+^/EIIIB^+^-containing fibronectin and require endothelial expression of either α5β1 or the αv integrins.

## Methods

### Mouse lines

All mice were housed and handled in accordance with approved Massachusetts Institute of Technology Division of Comparative Medicine protocols (IACUC approval 0412-033-15). *Fn* floxed [31]*, Itga5* floxed [26], *Itgav* floxed [32], Rosa26-CreER^T2^ [33], Cdh5(PAC)-CreER^T2^ [34], and mTmG [35], mouse lines have all been described previously. Live EIIIA/EIIIB KO mice were establish by extensively backcrossing EIIIA/EIIIB heterozygous mice [21] to C57BL/6 J wild-type mice and inter-crossing the resulting mice at *n* = 10. Cre activity and gene deletion were induced in *Fn* ^iKO^ and *Fn* ^iEC KO^ mice through consecutive intraperitoneal injections of 50 mg tamoxifen (Sigma) at P1, P2 and P3. Genotyping was performed on DNA isolated from tail snips in-house or by Transnetyx.

### Immunofluorescence staining

Whole-mount immunofluorescence staining of retinas was achieved following methods previously described in Pitulescu et al. [36]. Briefly, eyes were freshly isolated and fixed in 4% paraformaldehyde (PFA) in PBS at 4 °C overnight, blocked in PBS containing 0.5% Tween (PBS-T) and 2% goat, donkey or fibronectin-depleted goat serum and incubated overnight at 4 °C with primary antibodies either in 0.25% Tween/1% serum in PBS (staining buffer) or, for isolectin-B4 staining, in Pblec buffer (1% Triton X-100, 1 mM CaCl2, 1 mM MgCl2 and 1 mM MnCl2 in PBS, pH 6.8). After washes in PBS, retinas were incubated either at RT for 2 h, or overnight at 4 °C, with fluorophore-conjugated secondary antibodies diluted in staining buffer. Retinas were then washed in PBS, cut into three segments and flat-mounted onto coverslips in Fluoromount (Southern Biotech).

### Antibodies

Primary antibodies: Rat anti-mouse PECAM-1 MEC13.3 (1:100, BD Pharmingen), rabbit anti-Fibronectin (1:200, Hynes lab) [16], mouse anti-EIIIA fibronectin antibody (1:100, Abcam, IST-9), rabbit anti-Collagen IV (1:400, Abcam, ab19808), rabbit anti-Desmin (1:200, Abcam, ab15200), biotinylated Isolectin-B4 (1:50, Vector Labs, B-1205), mouse anti-EIIIB fibronectin (1:100, Amy McMahon, Hynes lab). Secondary antibodies were Alexa488, Alexa594, and Alexa647 conjugated antibodies (1:500, Invitrogen), and Alexa-Fluor–streptavidin-conjugated antibodies (1:200, Molecular Probes).

### Image acquisition and processing

All images were acquired using Zeiss LSM 510 or Nikon A1R scanning laser confocal microscopes and processed using Volocity (Perkin Elmer) or Nikon Elements software and Adobe Photoshop.

### Quantitative analyses of the retinal vasculature

All quantifications were completed using Volocity (Perkin Elmer) software using maximal intensity projection images.

Radial outgrowth/vessel migration was measured in a straight line from the optic nerve to the angiogenic front of the retinal plexus in 6 different mice from each genotype group (*n* = 6). Branch points were calculated from 250 μm × 500 μm fields of view (FOV) adjacent to retinal veins behind the angiogenic front using 6 different retina samples from each genotype group (*n* = 6).

Endothelial coverage, tip cell sprouts, filopodial numbers and lengths were all measured using only isolectin-B4 stained retinas. Endothelial percentage coverage is defined as the area of isolectin-B4-positive immunofluorescence divided by the total area, and was calculated from 340 mm^2^ fields within the capillary plexus behind the angiogenic front (*n* = 6 mice per genotype). Endothelial tip cell numbers were quantified by counting endothelial sprouts at the angiogenic front of the entire vascular plexus (*n* = 3 retinas per genotype). Endothelial tip cell filopodial numbers and lengths were calculated from high-resolution confocal images (60X objective, thin z-sections of sample) of 12 randomly selected tip cells at the leading edge of the vascular plexus from a minimum of 3 retinas per genotype (*n* = 12). Filopodia lengths were calculated from a minimum of 50 filopodia (*n* = 50).

Fibronectin deposition around the vasculature was quantified by measuring the mean fibronectin immunofluorescent pixel intensity in 10 randomly selected PECAM1-positive 500 μm^2^ fields within 200 μm of the leading edge of the capillary plexus, using a minimum of 2 retinas per genotype (*n* = 10). Vessel regression events were measured by counting PECAM1^−^/collagen IV^+^ structures within 6 FOV (sized 300 μm × 300 μm) adjacent to the retinal veins behind the angiogenic front using 3 retinas per genotype (*n* = 6). Endothelial cell proliferation was calculated by measuring the number of Ki-67^+^ PECAM1^+^ cells within 2 retinas and normalising to 100 μm vessel length (*n* = 2). Pericyte numbers were calculated from 6 fields (sized 230 μm × 230 μm) within the capillary plexus using a minimum of 3 retinas per genotype (*n* = 6).

Results are plotted as mean ± s.e.m and were analysed using Student’s *t* test and considered significant when *P < 0.05, **P < 0.01, ***P < 0.001 or ****P < 0.0001.

## Results

### Fibronectin controls the patterning of the retinal vasculature

**Fig. 1:**
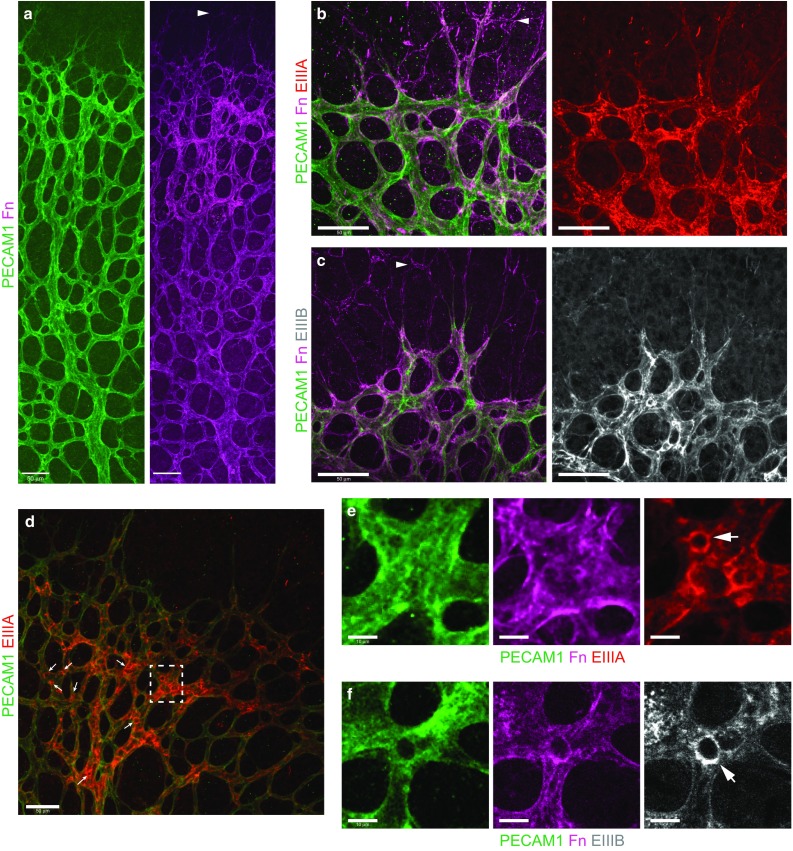
Fibronectin localisation within the retinal vasculature. **a–f** Whole-mount immunofluorescence staining showing the localisation of fibronectin and its alternatively spliced EIIIA^+^ and EIIIB^+^ -containing variants within the retinal vasculature of a P6 mouse. Fibronectin is localised throughout the vasculature (**a**) and is assembled just ahead of the vascular plexus by astrocytes (*arrowheads* **a–c**). EIIIA^+^ (**b**) and EIIIB^+^ (**c**) domains are present at low levels in the fibronectin network assembled by astrocytes (*arrowheads* **b, c**), but are found at higher levels in the fibronectin surrounding blood vessels. Note that expression of EIIIA (**d**, enlarged in **e**) and EIIIB (**f**) are especially pronounced around the edges of transcapillary pillars (*holes*) within vessels (*arrows*). *Scale bars*: 50 μm (**a–d**); 10 μm (**e, f**)

Fibronectin is highly expressed throughout the developing retinal vasculature (Fig. 1a–f) and is assembled ahead of the vascular plexus by retinal astrocytes (Fig. 1a–c) forming a scaffold for ECs migration [27, 37]. In contrast with the fibronectin assembled by astrocytes, the majority of the fibronectin surrounding the vasculature contained the alternatively spliced EIIIA and/or EIIIB domain(s) (Fig. 1b–f), with expression of both EIIIA and EIIIB especially pronounced around the edges of transcapillary pillars (holes) in vessels undergoing intussusceptive angiogenesis (Fig. 1d–f).

**Fig. 2:**
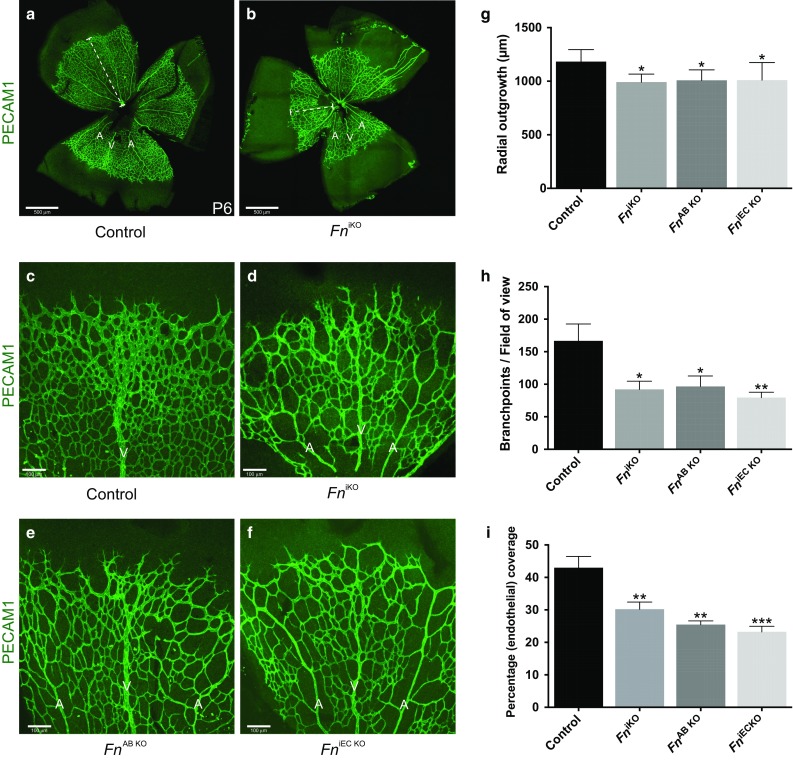
Abnormal patterning of the retinal vasculature in *Fn* mutants. **a–f** Confocal micrographs of the retinal vasculature in control, *Fn* ^iKO^, *Fn* ^AB KO^ and *Fn* ^iEC KO^ mice at P6. **a, b** Representative low magnification images showing that, in the absence of fibronectin, *Fn* ^iKO^ mice develop a vascular plexus containing arteries (*A*), veins (*V*) and capillaries, but display reduced vessel outgrowth (*dashed lines* illustrate measurements used to quantify distance of vessel migration), vascular branching and vascular coverage in their retinas (**b**, higher magnification **c**, **d**). These defects are phenocopied in *Fn* ^AB KO^ (**e**) and *Fn* ^iEC KO^ (**f**) mice. Quantification of vessel outgrowth (**g**), vessel branching (**h**) and endothelial coverage (**i**) (*see Methods*) reveals defects in the *Fn* mutants (*n* = 6, mice per genotype). *Scale bars*: 500 μm (**a, b**), 100 μm (**c–f**)

**Fig. 3:**
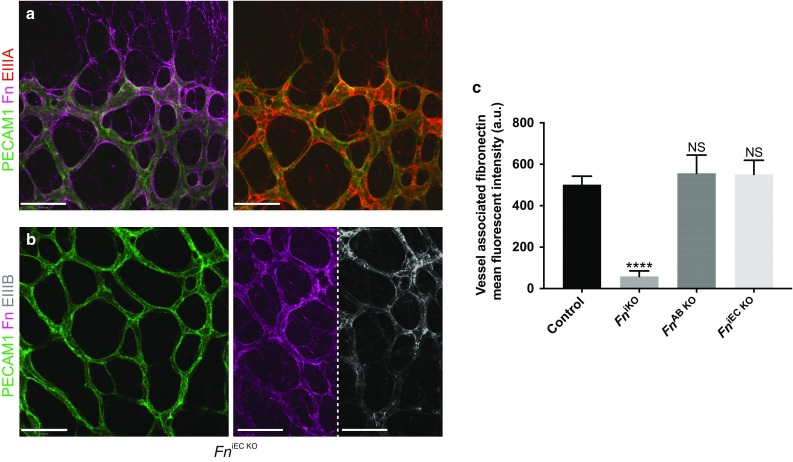
Deposition of fibronectin splice variants around the vessel wall of *Fn* ^iEC KO^ mice. Whole-mount immunofluorescence staining showing that *Fn* ^iEC KO^ mice develop vascular defects despite the presence of exogenous EIIIA^+^ (**a**) and EIIIB^+^ (**b**) fibronectin surrounding their vessels. **c** Quantification of fibronectin immunofluorescence around the retinal vasculature of *Fn* mutants showing that equivalent levels of fibronectin are deposited around the vessels of control, *Fn* ^iEC KO^ and *Fn* ^AB KO^ mice at P6 (*n* = 10, FOV). *NS* not significant. *Scale bars*: 50 μm

To investigate the role of fibronectin in regulating the development of the retinal vasculature we first deleted *Fn* from all postnatal tissue (*Fn* ^iKO^) by administering tamoxifen to newborn mice carrying the inducible (ubiquitously expressed) ROSA26-CreER^T2^ transgene and a loxP-flanked *Fn* gene (Fig. S1a). Surprisingly, despite previous studies showing that fibronectin is essential for early developmental angiogenesis [12, 18, 38, 39], whole-mount immunofluorescence staining of retinas revealed that both *Fn* ^flox/flox^ mice (control) and Rosa26-CreER^T2+^ *Fn* ^flox/flox^ mutants (*Fn* ^iKO^) develop a hierarchical vascular tree of arteries, veins and capillaries by postnatal day 6 (P6) (Fig. 2a–d). In contrast to control mice however, radial growth and vessel branching were significantly compromised in *Fn* ^iKO^ mice (Fig. 2a–f) and, as a consequence, endothelial coverage within the retinal tissue was significantly reduced (Fig. 2a–i). Since the EIIIA and EIIIB domains of fibronectin have been shown to regulate vascular morphogenesis [21], we next analysed the retinal vasculature in a rare family of surviving C57BL/6 EIIIA^−^ EIIIB^−^ double-knockout mice (*Fn* ^AB KO^). These mice express fibronectin around their vasculature at equivalent levels to control mice, however the Fn expressed no longer contains the EIIIA and EIIIB domains (Fig. S1b). Interestingly, phenocopying the *Fn* ^iKO^ mutants, *Fn* ^AB KO^ mice also displayed reduced vessel migration, branching and coverage within their retinas at P6 (Fig. 2e–h). Because EIIIA/EIIIB-containing fibronectin was predominantly localised around the developing vasculature in control retinas (Fig. 1b–f), and branching defects have not been reported in mice lacking astrocyte-derived fibronectin [27], we next examined whether the defects in both the *Fn* ^iKO^ and *Fn* ^AB KO^ could be due to the loss of just EC-derived EIIIA^+^/EIIIB^+^ fibronectin. To avoid embryonic developmental defects, we once again used an inducible loss-of-function approach and deleted *Fn* expression specifically in ECs from P1 onwards through the administration of tamoxifen to mice carrying a Cdh5(PAC)-CreER^T2^ transgene [34] and loxP-flanked *Fn* gene (*Fn* ^iEC KO^). Just as observed in *Fn* ^iKO^ and *Fn* ^AB KO^ mice, radial expansion, branching and density of retinal blood vessels were all significantly reduced in *Fn* ^iEC KO^ mutants (Fig. 2f, g–i). However, in contrast to both *Fn* ^iKO^ and *Fn* ^AB KO^ mutants (Fig. S1), and despite efficient Cre-mediated excision from endothelial cells (Fig. S2), blood vessels in *Fn* ^iEC KO^ mice remained covered with EIIIA^+^/EIIIB^+^ fibronectin at similar levels as observed in controls (Fig. 3a–c). This supports previous data showing that the majority of the fibronectin deposited around the vasculature in the retina is derived from astrocytes [27, 37], but suggests that astrocyte fibronectin cannot compensate fully for the loss of endothelial-derived fibronectin. Vessel patterning in the retina therefore is regulated, at least in part, in an autocrine manner by endothelial EIIIA^+^/EIIIB^+^ fibronectin.

### Endothelium-derived fibronectin affects tip cell numbers in the retina

**Fig. 4:**
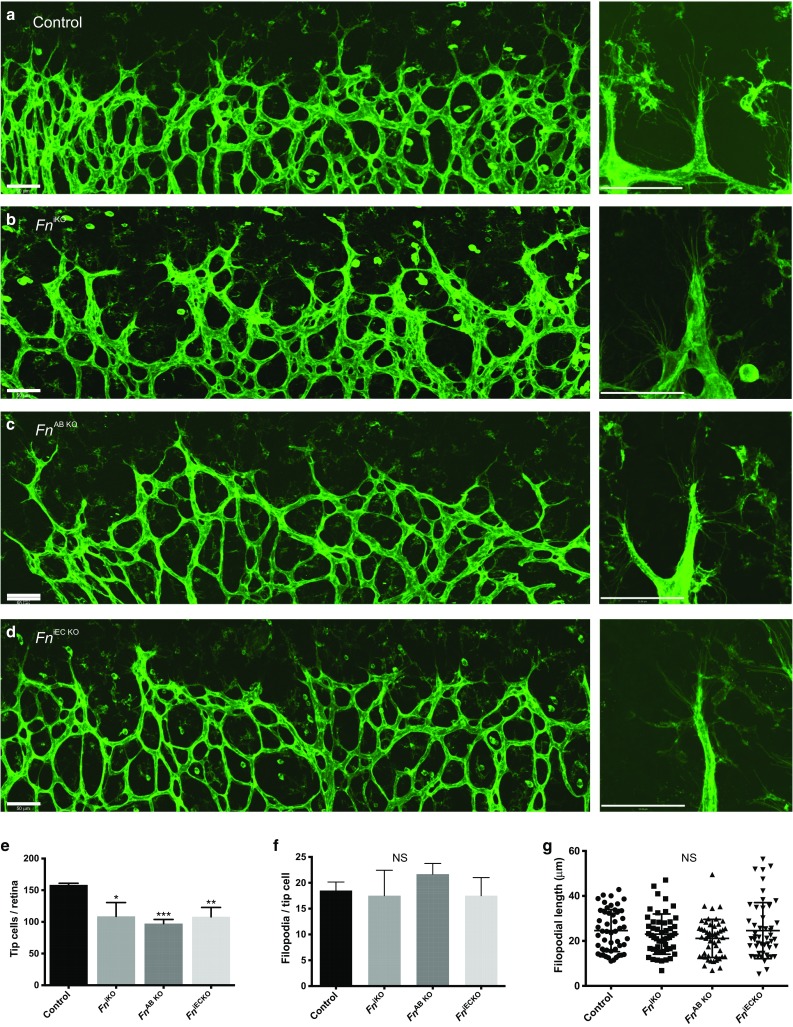
*Fn* mutants display reduced numbers of tip cells. Confocal images of isolectin-B4-stained tip cells at the angiogenic front of (**a**) control, (**b**) *Fn* ^iKO^, (**c**) *Fn* ^AB KO^ and (**d**) *Fn* ^iEC KO^ retinas at P6. *Fn* mutants have reduced numbers of extending tip cell vessel sprouts (*n* = 3, mice per genotype) (**e**), but have similar numbers of filopodial extensions (*n* = 12, tip cells) (**f**) and filopodial lengths (*n* = 50, filopodia) (**g**) per tip cell as control mice. Higher magnification images of individual tip cells and filopodial extensions in right panels. Note the thicker more irregular tip cell morphology in *Fn* ^iKO^ mutants (**a–d**). *NS* not significant. *Scale bars*: 50 μm

Examination of the leading edge of the vascular plexus revealed that the reduced vessel density in all three *Fn* mutants is, in part, due to a reduced number of tip cells at the angiogenic front of the retinal vasculature (Fig. 4a–e). In addition, global loss of fibronectin also led to increased numbers of thick, long, abnormally shaped angiogenic sprouts at the front of the plexus (Fig. 4a–d). Loss of fibronectin did not appear however to affect either the number or length of filopodia extending from individual tip cells in *Fn* mutants (Fig. 4f, g). In addition, filopodia extending from endothelial cells in *Fn* ^iEC KO^ mutants aligned with the fibronectin network assembled ahead of the plexus by the astrocytes (Fig. 3a).

### Endothelium-derived fibronectin regulates vessel stability

**Fig. 5:**
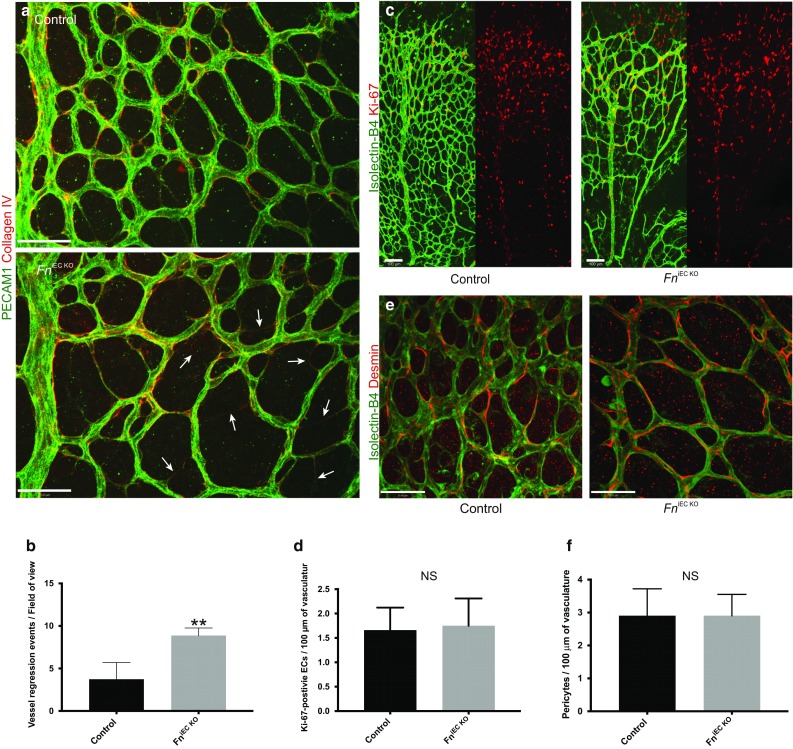
Loss of EC-derived fibronectin leads to ectopic vessel regression. **a** *Fn* ^iEC KO^ mice display increased numbers of thin, empty (PECAM1-negative) sleeves of collagen IV matrix (*arrows*), indicating increased levels of vessel regression within their retinas at P6. **b** Quantification of PECAM1^−^ collagen IV^+^ vessel regression segments (*n* = 6, FOV). **c** Immunofluorescence staining for the proliferation marker Ki-67, and (**d**) quantification of proliferating endothelial cells, revealed no significant differences in endothelial cell (EC) proliferation within the retinas of *Fn* ^iEC KO^ mice at P6 (*n* = 2, mice per genotype). **e** Desmin-positive pericytes remain in close association with the capillary endothelium in *Fn* ^iEC KO^ mice, and are found in similar numbers to those seen in control mice (**f**) (*n* = 6, FOV). *Scale bars*: 50 μm (**a, e**); 100 μm (**c**)

Further analysis revealed that EC-derived fibronectin also has a critical role in controlling vessel stability. At P6, *Fn* ^iEC KO^ mutants had increased numbers of thin empty PECAM1-negative/collagen IV-positive basement membrane sleeves, which are left behind by regressing endothelial cells and serve as a historical record of pre-existing vessels [40, 41], indicating increased pruning/regression of vessels (Fig. 5a, b). Proliferation of endothelial cells in *Fn* ^iEC KO^ mutants however appeared largely unaffected by the loss of fibronectin (Fig. 5c, d). Previous studies have shown that vessel stability is dependent on the recruitment of mural cells [42], namely pericytes and vascular smooth muscle cells. We therefore analysed whether loss of EC-derived fibronectin inhibited the recruitment and incorporation of mural cells around retinal vessels. Analysis of P6 retinas stained with anti-desmin antibodies however revealed no obvious defects in mural cell recruitment or attachment to the capillaries of *Fn* ^iEC KO^ mutants (Fig. 5e, f).

### Mice lacking endothelial α5 and αv integrins phenocopy *Fn*^iEC KO^ mutants

**Fig. 6:**
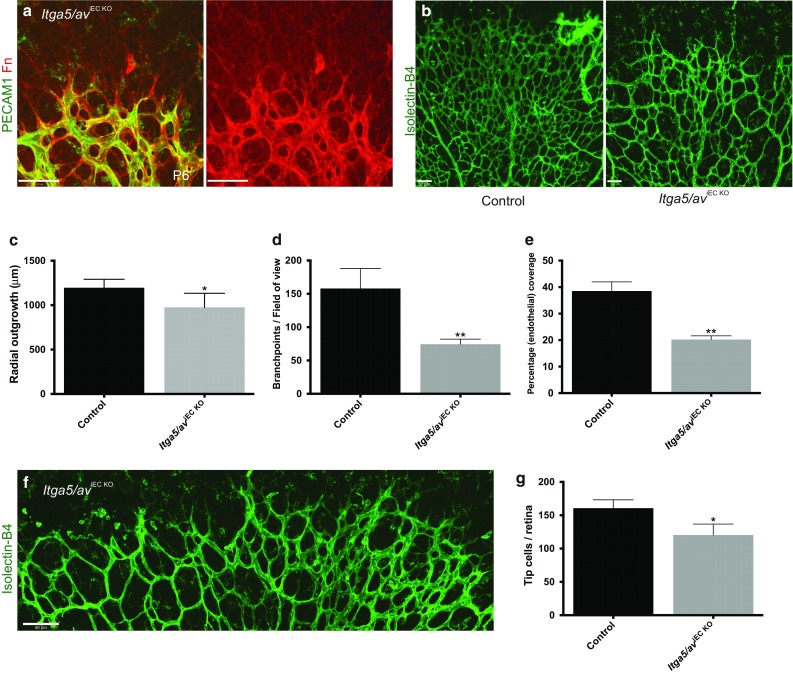
Loss of both endothelial α5 and αv integrins phenocopies *Fn* mutants. **a** Immunofluorescence staining showing deposition of fibronectin around the retinal vasculature of an *Itga5/av* ^iEC KO^ mouse at P6, despite the lack of the major fibronectin-binding integrins. **b** Isolectin-B4-labeled retinas showing the decreased vessel branching and density in *Itga5/av* ^iEC KO^ retinas. Quantification of (**c**) radial outgrowth, (**d**) branchpoints and (**e**) endothelial coverage in *Itga5/av* ^iEC KO^ mutants (*n* = 6, mice per genotype). **f** Representative image of the tip-cell sprouts at the angiogenic front of the developing vasculature in *Itga5/av* ^iEC KO^ mice. **g** Quantification of *Itga5/av* ^iEC KO^ tip-cell deficit (*n* = 3, mice per genotype). *Scale bars*: 50 μm (**a, b, f**)

Since *Fn* ^iEC KO^ mice displayed vascular defects, despite displaying apparently normal levels of cellular fibronectin around their vessels (Fig. 3), we next investigated whether vascular patterning in the retina is dependent on expression of the major endothelial fibronectin-binding receptors, integrin α5 and αv. Just like fibronectin, α5 and αv integrins are poorly expressed on quiescent endothelium but are highly expressed around blood vessels during developmental or tumour angiogenesis. Previous studies have shown that mice lacking endothelial expression of both α5 and αv die at E14.5 with heart, great vessel and lymphatic defects [26, 43] but, in contrast to numerous in vitro studies, lack angiogenic defects [26]. To examine the role of endothelial α5 and αv integrins in postnatal developmental angiogenesis, we crossed female double-homozygous *Itga5*/*Itgav*-floxed mice to Cdh5(PAC)-CreER^T2^ mice (to generate *Itga5/av* ^iEC KO^ mice) and deleted both genes from the endothelium through administration of tamoxifen from P1 to bypass embryonic lethality. Despite the requirement for both α5 and αv integrins for fibronectin fibrillogenesis in vitro, consistent with previous in vivo studies [25, 26], *Itga5/av* ^iEC KO^ mice displayed no obvious defects in the assembly of Fn around their vasculature (Fig. 6a). However, just as observed in *Fn* ^iEC KO^ mutants, doubly deficient *Itga5/av* ^iEC KO^ (but not singly deficient *Itga5* ^iEC KO^ or *Itgav* ^iEC KO^ mice, data not shown) still displayed reduced radial growth, vessel branching, endothelial coverage, and tip cell numbers within their retinas at P6 (Fig. 6b–g). Surprisingly, in contrast to mice in which *Itga5* had been deleted from the endothelium using Tie2-Cre [27], endothelial tip-cell filopodia appeared unaffected by the loss of both α5 and αv integrins and aligned to the fibronectin network assembled by astrocytes (Fig. 6a). The possibility exists that other fibronectin-binding integrins could also be involved in retinal angiogenesis or that they might partially compensate for the absence of α5 and αv. Nonetheless the results do show the involvement of these two integrin subunits.

## Discussion

In this study, we have shown that vessel patterning in the retina is regulated in an autocrine manner by EC-derived EIIIA^+^/EIIIB^+^ fibronectin and that these functions are not fully compensated by exogenous sources of fibronectin such as astrocytes or plasma. We have also shown that postnatal developmental angiogenesis requires endothelial expression of at least one of the RGD-binding integrin receptors and that interaction of α5 and αv integrins with EC-derived fibronectin regulates vessel sprouting and vessel stability during vascular development.

### Fibronectin regulates blood vessel development

It has long been suggested that fibronectin plays a key role in the development of the vascular system. Fibronectin is found only in vertebrates with an endothelial-lined vasculature [10] and forms a central node within the “angiome” [44]. The exact functions of Fn and its splice variants during vascular development however have remained unclear due to mesodermal, neural tube and cardiac defects hampering the interpretation of the vascular defects observed in existing *Fn* models [12, 17, 18, 21]. Our detailed analyses of retinal vascular development show that, in the absence of embryonic defects, mice temporally lacking *Fn* expression throughout the tissue develop severe vascular patterning defects (Fig. 2). *Fn* ^iKO^ mice are able to develop vessels, but radial expansion, vessel branching and density of blood vessels are all reduced in the absence of *Fn* (Fig. 2). While the reduced radial outgrowth of blood vessels can in part be attributed to the loss of astrocyte-derived Fn within the retina and reduced VEGFR2 and PI3 K/Akt signalling [27], our results indicate that it is the loss of EC-derived *Fn* that is the predominant cause of the vascular defects observed. *Fn* ^iEC KO^ mutants display decreased vessel outgrowth, branching, and vessel coverage, phenocopying the defects observed in global *Fn* KO mice (Fig. 2). These defects are, at least in part, due to decreased levels of vessel sprouting at the angiogenic front (Fig. 4) and increased regression and pruning within the vascular plexus (Fig. 5). Since Fn expression is especially pronounced around the edges of transcapillary pillars, we cannot however rule out the possibility that it may also regulate intussusceptive angiogenesis, which has also been shown to increase vessel branching and vascular expansion in numerous tissues including the retina [45].

### Cell autonomous roles for fibronectin during vascular development

A remarkable finding in our study is that *Fn* ^iEC KO^ mice develop vascular defects despite displaying apparently normal levels of Fn around their vessels (Fig. 3), suggesting that EC-derived Fn plays a distinct role in regulating vascular development. During the development of the retina, *Fn* is expressed by ECs, astrocytes [27, 37] and, to some extent, pericytes [46], although the functional importance of the latter appears to be minimal since no defects have been observed in mice lacking pericyte expression of *Fn* (data not shown). Astrocytes deposit a Fn scaffold ahead of the vascular plexus to support EC migration into the avascular areas of the retina (Fig. 1), but upon contact with the advancing vasculature astrocytes downregulate fibronectin expression [27, 37]. Interestingly, in contrast to *Fn* ^iEC KO^ mutants, astrocyte-specific deletion of *Fn* leads to increased numbers of tip-cell filopdia, increased branching and an increase in vessel density [27]. Taken together, this suggests that Fn regulates vascular development through distinct paracrine and autocrine mechanisms, with astrocyte Fn inhibiting and EC-derived Fn promoting sprouting and branching of vasculature. Autocrine fibronectin has previously been suggested to have a distinct role in controlling EC behaviour in vitro [29], and has been shown to play an important role in regulating cardiovascular development in vivo [47]. The exact mechanisms by which autocrine Fn elicits its differential response however remain unclear.

### Roles of EIIIA/EIIIB-containing fibronectin

One possible hypothesis for the differential response to paracrine and autocrine Fn is that ECs may produce a different form of fibronectin. Fn containing EIIIA/EIIIB domains is highly expressed around the developing vasculature, but these domains are almost undetectable in the Fn scaffold deposited by astrocytes in the retina (Fig. 1). Furthermore, loss of both EIIIA and EIIIB domains, but of neither one alone (data not shown), replicates the vascular defects observed in the *Fn* ^iEC KO^ mutants (Figs. 2, 4). Arguing against this hypothesis however is the observation that *Fn* ^iEC KO^ mutants still display EIIIA- and EIIIB-containing fibronectin around their vessels (Fig. S1b). A caveat of this analysis however is that it is almost impossible to distinguish whether the Fn surrounding the vessels is EIIIA^+^ EIIIB^+^ or just fibrils of Fn containing a mixture of EIIIA^+^ EIIIB^−^ and EIIIA^−^ EIIIB^+^ fibronectins. Since neither EIIIA KO nor EIIIB single KO mice display any vascular defects [19], it is possible that only EIIIA^+^ EIIIB^+^ Fn is expressed by ECs, and it is this specific form of Fn that is essential for regulating EC function. So, how might the EIIIA and EIIIB domains regulate EC function? Previous studies have shown that Fn can bind and regulate the activity of numerous growth factors [48], it is possible therefore that the addition of EIIIA and EIIIB domains may modulate growth factor signalling within the vascular endothelium. Indeed, EIIIA and EIIIB KO cells have reduced growth and proliferation in vitro [20, 49]. It is unlikely that this is the cause of the vascular phenotype seen in *Fn* ^iEC KO^ mice however, since no obvious proliferation defects were observed in our mutant mice (Fig. 5). A second possibility is that the addition of EIIIA and EIIIB domains may alter the physiological properties of Fn. Fibronectin is one of the most extendable biological fibres [50, 51] and upon extension becomes more rigid [52]. It is conceivable therefore that addition of EIIIA and EIIIB may alter the structural and mechano-transductive properties of the protein. Fn fibrillogenesis [53], assembly of collagen I [54], and vasodilation of vessels [55] have all been shown to be mechano-regulated by stretch-induced conformational changes in Fn. Finally, insertion of additional EIIIA and EIIIB domains may increase adhesiveness of Fn to its integrin receptors. The EIIIA domain contains additional binding sites for α4β1 and α9β1 integrins [56], while inclusion of EIIIB has been shown to induce a conformational change that unmasks a cryptic binding site [57] and affects the exposure of the RGD loop [58] recognised by both α5 and αv integrins within Fn.

### Role of α5 and αv integrins during vascular development

In contrast to studies in the embryo [26] and during tumorigenesis [30], we found that endothelial expression of both α5 and αv integrins is essential for proper angiogenesis in the retina (Fig. 6). Just as observed in both *Fn* ^iEC KO^ and *Fn* ^AB KO^ mutants, loss of both α5 and αv integrins leads to defects in vessel growth, branching, and vascular sprouting (Fig. 6). Interestingly, similar defects have been reported in mice lacking EC expression of integrin β1 [7], suggesting that interaction of EC-derived EIIIA^+^ EIIIB^+^ Fn with α5β1 and αvβ1 regulates vessel patterning and stability during retinal angiogenesis. We have previously shown that α5 and αv integrins cooperate to regulate vascular smooth muscle cell function in vivo [25], it is therefore increasingly clear that both receptors play a key role(s) in controlling the development of the vascular system. These results do not rule out the participation of other integrins recognising fibronectin or, indeed, other ECM proteins in retinal angiogenesis.

## Conclusion

Very few prior studies have given much attention to the cellular sources or specific splice variants of Fn within their experiments. Our results have shown that EC-derived Fn provides distinct signals from those derived from exogenous sources of Fn (such as astrocytes, pericytes, and the plasma) and is indispensible for proper vascular development in the retina. Furthermore, we have shown that EC-derived fibronectin requires both EIIIA and EIIIB domains for its function and that it signals through α5 and αv integrins to regulate vessel patterning. It is quite possible that detailed analyses of other angiogenic processes may reveal analogous distinctions among the contributions of different cell types and isoforms to specific aspects of angiogenesis. The exact mechanisms by which EIIIA and EIIIB domains within Fn regulate angiogenesis and the precise roles of the individual Fn integrins on vascular cells however remain unclear. Future experiments will need to examine the biomechanical and biochemical signalling changes caused by the addition of EIIIA and EIIIB domains within fibronectin and use multiple inducible cell-specific integrin and Fn mutants. It will also be important to identify the genetic modifiers that strongly influence the phenotypes of both integrin and fibronectin mutants.

## Electronic supplementary material

Below is the link to the electronic supplementary material.

Supplementary Fig. 1 Fibronectin deposition in *Fn* ^iKO^ and *Fn* ^AB KO^ mutants. Whole-mount immunofluorescence staining confirming (**a**) loss of fibronectin (middle and right panels) in *Fn* ^iKO^ retinas by P6 following tamoxifen injections from P1-P6, and (**b**) loss of EIIIA expression (left panel), but normal levels of EIIIA/EIIIB^−^ fibronectin (right panel, magenta) around the vasculature in *Fn* ^AB KO^ mice. Scale bars: 50 μm. (AI 14579 kb)

Supplementary Fig. 2. Confirmation of efficient gene excision in *Fn* ^iEC KO^ mice. Confocal micrograph showing efficient Cdh5(PAC)-CreER^T2^ mediated activation of the mTmG reporter, in which Cre-mediated excision results in the expression of membrane-bound GFP, in endothelial cells within the retina of a *Fn* ^iEC KO^ mice at P6, following three consecutive tamoxifen injections (from P1-3). Scale bar: 50 μm. (AI 8089 kb)
